# Watch-Sensed Socialization and Cognitive Task Performance for Older Adults in a Rural Community

**DOI:** 10.1093/geroni/igaf122.1598

**Published:** 2025-12-31

**Authors:** Diane Cook, Bryan Minor, Christine Williams, Janet Holt, Juyoung Park, Lilah Besser, Diana Mitsova, Lisa Wiese

**Affiliations:** Washington State University, Pullman, Washington, United States; Washington State University, Pullman, Washington, United States; Florida Atlantic University, Boca Raton, Florida, United States; Florida Atlantic University, Boca Raton, Florida, United States; University of Arizona, Tucson, Arizona, United States; University of Miami, Boca Raton, Florida, United States; Florida Atlantic University, Boca Raton, Florida, United States; Florida Atlantic University, Boca Raton, Florida, United States

## Abstract

An extensive body of research links social isolation, physical inactivity, and rural residence with cognitive decline. The long-term goal of this work is to analyze the contribution of PM2.5 exposure during agricultural burn seasons to social isolation and cognitive function. As a step toward this goal, we investigated how smartwatch-based measures of socialization and physical activity relate to cognitive performance in older multicultural adults living in a rural southern Florida farmworker region. A total of n = 36 older adult participants (29 female, 7 male, mean age = 64.28) wore Apple Watches for two weeks to track movement and location, while cognitive function was assessed daily using a 45-second n-back shape test delivered via the watch. We quantified socialization as the percentage of time spent outside the home and defined activity level as the mean magnitude of the acceleration vector. Daily markers for socialization and activity level were extracted. Given individual differences in cognitive performance, we calculated daily deviations from each participant’s mean n-back score. We then assessed correlations between these cognitive deviations and behavior markers. Across 798 data points, we found a small but statistically significant correlation between n-back score deviations and time spent outside the home (r = 0.190, p <.001). In contrast, the correlation between n-back score deviations and physical activity level was not significant (r=.033, p=.354). This ongoing work highlights the potential of continuous sensing to evaluate the influence of environmental and social factors on ADRDs and design community-specific interventions to promote brain health.

